# Enhanced anti-hyperuricemic effect of Simiao pill by composite lactic acid bacteria fermentation: ferulic acid and berberine enrichment and URAT1/OAT1/XOD regulation

**DOI:** 10.3389/fphar.2026.1801987

**Published:** 2026-04-30

**Authors:** Xia Zeng, Yi Huang, Guangying Luo, Siqi You, Jia Li, Yong Jiang, Zhengwen Li, Zhengyou He, Sichong Ren

**Affiliations:** 1 Laboratory of Chinese Medicinal Resources and New Product Development, School of Pharmacy, Sichuan Industrial Institute of Antibiotics, Chengdu University, Chengdu, China; 2 Department of Nephrology, Clinical Medical College of Chengdu Medical College, Chengdu, China

**Keywords:** berberine, composite lactic acid bacteria, fermentation, ferulic acid, hyperuricemia, simiao pill

## Abstract

**Background:**

Simiao Pill (SP) is a traditional Chinese medicine used to treat hyperuricemia (HUA). Microbial fermentation can enhance the effectiveness of herbal medicines.

**Objectives:**

This study aimed to evaluate the effects of composite lactic acid bacteria fermentation on the chemical components of SP and its anti-HUA effect.

**Methods:**

This study revealed the contents of fermented Simiao Pill (FSP), and optimized the optimal fermentation conditions. The anti-HUA effects were evaluated by testing the biochemical indicators of liver and kidney function, and the histology of liver and kidney. Immunohistochemistry, Western blot and RT-qPCR analysis were applied to investigate key proteins and mRNAs.

**Results:**

After fermentation with composite lactic acid bacteria, the contents of ferulic acid and berberine in fermented Simiao Pill (FSP) were increased by 40.0% and 20.7%, respectively, compared with those in SP. The optimal fermentation conditions were: time of 24 h, temperature of 35 °C, and inoculation of 1%. FSP significantly reduced the serum levels of uric acid, blood urea nitrogen, creatinine. FSP ameliorated the damage of the kidney and the degree of renal interstitial fibrosis. FSP downregulated the protein expressions of URAT1, upregulated that of OAT1, and inhibited the expression of XOD activity and protein, with its efficacy being superior to that of SP.

**Conclusion:**

Fermentation with composite lactic acid bacteria increased the contents of ferulic acid and berberine in SP. FSP lowered uric acid by increasing OAT1 to promote uric acid excretion, decreasing URAT1 to reduce uric acid reabsorption, and inhibiting XOD to reduce uric acid production.

## Graphical Abstract

**Figure:**
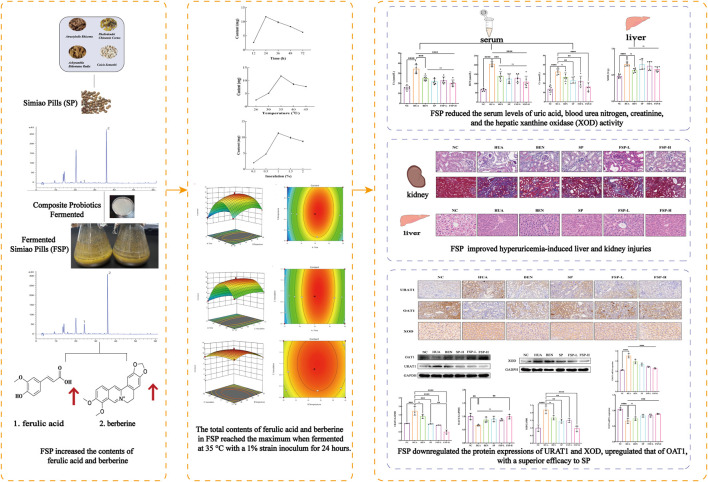

## 1 Introduction

Hyperuricemia (HUA) is a metabolic disorder caused by poor regulation of purine metabolism (Benn et al., 2018). HUA has become known as the fourth common basic metabolic disease after hypertension, hyperglycemia, and hyperlipidemia. And HUA is recognized as the second most common metabolic disease after diabetes (Li et al., 2020b). Current treatments for HUA include drugs that inhibit uric acid synthesis and those that promote uric acid excretion, with the latter being a primary therapeutic approach (Li et al., 2020b). Western medicines such as allopurinol (Wang et al., 2023), febuxostat (Shang et al., 2023), and benzbromarone (Kohagura et al., 2023) are commonly used, but these may cause side effects like stomach irritation and liver or kidney damage. In contrast, traditional Chinese medicine takes a holistic approach, using herbs that clear heat, remove dampness, and activate blood flow to treat HUA and related conditions such as gout (Lin et al., 2016).

Simiao Pill (SP) comes from the Qing Dynasty text *Cheng Fang Bian Du.* SP consists of *Atractylodis Rhizoma*, *Phellodendri Chinensis Cortex*, *Achyranthis Bidentatae Radix*, and *Coicis Semen*. This formula clears heat, removes dampness, eliminates stagnation, and unblocks meridians. SP is often used in clinical practice to treat HUA and gout (Zeng et al., 2024). Previous studies have demonstrated that SP promotes uric acid excretion by regulating the expression of urate anion transporter 1 (URAT1) and organic anion transporter 1 (OAT1), and inhibits xanthine oxidase (XOD) activity (Hu et al., 2010). Furthermore, the primary active components of SP, including ferulic acid and berberine, contribute to its uric acid-lowering effects by inhibiting XOD (Lou et al., 2023) activity and reducing monosodium urate deposition (Dinesh and Rasool, 2017).

Fermentation is a key processing technique in traditional Chinese medicine that can enhance therapeutic efficacy (Li et al., 2020a). For instance, Cui et al. (2025) demonstrated that fermentation of Sophora japonica flowers with composite lactic acid bacteria increased the content of total flavonoids, phenolics, alcohols, and esters, resulting in enhanced antioxidant activity. Similarly, Wang et al. (2024a) found that fermentation of *Astragalus membranaceus* with composite lactic acid bacteria increased the yield of astragaloside IV. Moreover, fermented Astragalus extracts significantly activated antioxidant signaling pathways, promoting the expression of antioxidant genes such as nuclear factor erythroid 2-related factor 2, heme oxygenase 1, NAD(P)H quinone dehydrogenase 1, and superoxide dismutase 1, and provided protective effects against lipopolysaccharide-induced oxidative damage in Normal Human Colon Mucosal Epithelial Cells compared to unfermented extracts.

Our research group previously investigated the fermented Simiao Pill (FSP) with various microbial strains and identified that composite probiotic fermentation most significantly increased the content of its primary active components, ferulic acid and berberine. To compare the changes in active components of SP before and after fermentation and to assess whether FSP exerts superior uric acid-lowering effects, this study investigated the changes in active components before and after fermentation of SP with composite lactic acid bacteria and conducted optimization experiments for its fermentation conditions. By establishing a HUA mice model induced by potassium oxonate combined with adenine, this study evaluated uric acid levels, blood urea nitrogen, kidney injury repair, and liver toxicity in mice to investigate whether FSP exerts a uric acid-lowering effect and to elucidate the underlying mechanisms.

## 2 Materials and methods

### 2.1 Materials

The four herbal ingredients of SP were purchased from the Chengdu Hehuachi Chinese Herbal Medicine Market, Chengdu, China, and authenticated to belong to their respective botanical families. Glucose, MgSO_4_.7H_2_O, KH_2_PO_4_ were purchased from Chengdu Kelong Chemical Co., Ltd. The composite lactic acid bacteria, consisting of *Lactobacillus casei* (CGMCC 1.15608), *Lactobacillus plantarum* (CGMCC 1.12934), *Lactobacillus reuteri* (CGMCC 1.2838), *Lactobacillus salivarius* (CGMCC 1.1881), and *Lactobacillus acidophilus* (CGMCC 1.3342) in a 1:1:1:1:1 ratio, was obtained from the China General Microbiological Culture Collection Center (CGMCC), Beijing, China. These strains were subsequently maintained and subcultured in our laboratory. Ferulic acid was purchased from Chengdu Purifa Biotechnology. Berberine was purchased from Chengdu Desite Biotechnology. Adenine and Potassium Oxonate were purchased from Chengdu Kelong Chemical. Uric Acid (UA) Assay Kit, Blood Urea Nitrogen (BUN) Assay Kit, Creatinine (Cr) Assay Kit and Xanthine Oxidase (XOD) Assay Kit were purchased from Nanjing Jiancheng Bioengineering Institute. Xylene and 3% Hydrogen Peroxide were obtained from Shanghai Hushi Laboratory Equipment. Citric Acid Antigen Retrieval Solution and Hematoxylin Staining Solution were purchased from Nanjing Yiermei Biotechnology. Neutral Gum was purchased from Sinopharm Chemical Reagent. URAT1 Antibody and XOD Antibody were obtained from Chengdu Zhengneng Biotechnology. OAT1 Antibody was purchased from Bioss Antibodies. Anti-GAPDH was obtained from Affinity. Goat anti-Rabbit IgG was bought from BioPM. Anti-Xanthine Oxidase Rabbit mAb was provided by ABclonal Technology Co.,Ltd. Antibodies for URAT1, Collagen I, α-smooth muscle actin (α-SMA) and OAT1 were provided by Proteintech Group, Inc.

### 2.2 Preparation of SP

An appropriate anmount of medicinal materials including *Atractylodis Rhizoma*, *Phellodendri Chinensis Cortex*, *Achyranthis Bidentatae Radix*, and *Coicis Semen* was taken, pulverized, and sieved through a 40-mesh sieve for subsequent use. Then, in accordance with the specifications of the *Pharmacopoeia of the People’s Republic of China* (2020), 4 g of *Achyranthis Bidentatae Radix*, 4 g of *Atractylodis Rhizoma*, 8 g of *Coicis Semen*, and 8 g of *Phellodendri Chinensis Cortex* were weighed and mixed thoroughly to obtain the powder mixture of Simiao Wan.

### 2.3 Cultivation of composite lactic acid bacteria

Resuscitation of Frozen Strains: The frozen strains of *Lactobacillus casei*, *Lactobacillus plantarum*, *Lactobacillus reuteri*, *Lactobacillus salivarius*, and *Lactobacillus acidophilus* were revived. The liquid medium for composite lactic acid bacteria: 200 g/L potato, 20 g/L glucose, 1.5 g/L MgSO_4_·7H_2_O, 3 g/L KH_2_PO_4_, 0.02 g/L vitamin B_1_. The medium was then sterilized at 121 °C for 30 min. After cooling, the five bacterial strains were inoculated into the medium at a 1:1:1:1:1 ratio to form the composite lactic acid bacteria (Wei et al., 2026; Li et al., 2017). The culture was subcultured for four consecutive generations.

### 2.4 Preparation of FSP

Added 24 g SP powder to 250 mL of liquid medium in a sealed shake flask, and sterilized at 121 °C for 30 min. After cooling to room temperature, the medium was inoculated with 1 × 10^10^ CFU/mL of the fourth-generation composite lactic acid bacteria and incubated at 35 °C for 24 h. The fermentation broth was then centrifuged at high speed, filtered, and concentrated to obtain the extract for subsequent use.

### 2.5 Determination of component changes before and after fermentation using HPLC

#### 2.5.1 Preparation of test sample solutions

Aliquots (5 mL) of blank culture medium, FSP solution, and SP solution were each transferred into 25 mL volumetric flasks and diluted to volume with methanol. The samples were then sonicated for 20 min, filtered, and the filtrates were collected for subsequent analysis.

#### 2.5.2 Preparation of reference standard solutions

Ferulic acid and berberine reference standards were dissolved in methanol to prepare solutions with approximate concentrations of 0.15 mg/mL for ferulic acid and 0.50 mg/mL for berberine, respectively.

#### 2.5.3 HPLC conditions

The analysis was performed using a SWELL C18 column (250 mm × 4.6 mm, 5 µm). The column temperature was maintained at 30 °C, with a flow rate of 1 mL/min and an injection volume of 10 µL. The detection wavelength was set at 280 nm. Specific elution parameters were detailed in Table 1 (Gou et al., 2022).

**TABLE 1:** HPLC elution conditions.

Time (min)	A (acetonitrile)	B (0.1% phosphoric acid)
0	7%	93%
30	24%	76%
40	51%	49%
60	70%	30%
90	90%	10%

### 2.6 Fermentation condition optimization test

Single factor tests were carried out on the fermentation temperature (26, 30, 35, 40, 45 °C. Fermented with 1.5% inoculation for 24 h), inoculation (0.1, 0.5, 1.0, 1.5, 2.0%. Fermentation at 35 °C for 24 h), and termination fermentation time (12, 24, 36, 48, 72 h. Under the conditions of 1.5% inoculation and 35 °C). Then, the three factors were selected for the Box–Behnken response surface optimization test using the Design Expert 13 (Table 2). Similarly, the total content of ferulic acid and berberine in FSP was used as the response value.

**TABLE 2:** Level table of experimental design factors of response surface.

Levels	Factors		
	A Time (h)	B Temperature (°C)	C Inoculation (%)
−1	12	30	0.5
0	24	35	1.0
1	36	40	1.5

### 2.7 Experimental animals

The animals used in this study were specific pathogen-free (SPF) male C57 mice (weight: 20 ± 2 g, age: 6–8 weeks), totaling 60 mice, provided by Chengdu Dashuo Experimental Animal, with an animal production license number SCXK (Chuan) 2020-030. The mice were housed under controlled conditions with a temperature of 24 °C ± 2 °C, relative humidity of 50%–65%, and a 12 h light/dark cycle. They were kept in separate cages with *ad libitum* access to food and water throughout the experiment. They were examined by the Animal Welfare Ethics Committee of The First Affiliated Hospital of Chengdu Medical College (No. 2023CYFYIRB-BA-Dec01).

### 2.8 Animal grouping, modeling and dosages

Sixty C57 mice were randomly divided into six groups (n = 10 per group): normal control (NC), HUA, benzbromarone (BEN), SP, fermented Simiao Pill low-dose (FSP-L), and fermented Simiao Pill high-dose (FSP-H). Except for the NC group, all other groups were induced to develop a HUA model through oral administration of 200 mg/kg potassium oxonate combined with 75 mg/kg adenine (Zhang et al., 2018), once daily. On the 7^th^ day of modeling, 1 mL of blood was collected from the orbital plexus under anesthesia, allowed to stand at room temperature for 1–2 h until coagulation, and then centrifuged at 4 °C and 3,000 rpm for 10 min. The supernatant was collected, and UA levels were measured according to the instructions of the UA assay kit. Starting on the 8^th^ day, based on referenced literature (Ogino et al., 2019), the Chinese Pharmacopoeia (2020 edition), and the clinical dose for humans combined with the body surface area conversion formula for mice (assuming the body weight of an adult is 70 kg, the conversion coefficient for mouse body surface area is 0.0025), the treatment groups were administered the following drugs via oral gavage: the positive control group received 6.5 mg/(kg·d) BEN, SP and FSP-H groups received 1.48 g/(kg·d), and the FSP-L group received 0.35 g/(kg·d). The NC and HUA groups were administered an equivalent volume of normal saline. Treatments were administered once daily for 27 consecutive days (Figure 4A). In addition, their body weight was recorded every 7 days. During this period, the mice’s mental status, motor activity, and food intake were observed daily.

### 2.9 Sample collection and processing

Following the final administration, mice were fasted for 12 h, and their body weights were recorded. All groups were anesthetized via intraperitoneal injection of sodium pentobarbital (80 mg/kg). Blood was collected from the orbital plexus and allowed to stand at room temperature for 1–2 h to coagulate. Subsequently, the blood samples were centrifuged at 3,000 rpm for 10 min at 4 °C to obtain serum, which was stored at −80 °C. Bilateral renal and hepatic tissues were harvested from the mice and preserved at −80 °C for subsequent analysis.

### 2.10 Biochemical analysis

The levels of UA, BUN, Cr, AST and ALT in mice serum and XOD in the liver were determined using corresponding kits, following the manufacturer’s instructions.

### 2.11 Histopathological and immunohistochemical analysis of renal and hepatic tissues

Renal and hepatic tissues were rinsed with phosphate-buffered saline (PBS) and fixed in 4% paraformaldehyde solution for 24 h. The tissues were then dehydrated, embedded in paraffin, and sectioned. Sections were stained with hematoxylin-eosin for 5 min, dehydrated, and sealed with neutral gum. Histopathological changes were observed under an optical microscope, and images were captured for analysis.

Paraffin-embedded sections were deparaffinized in xylene and rehydrated through a graded ethanol series. Antigen retrieval was performed by immersing the sections in pH 6.0 citrate buffer. To eliminate endogenous peroxidase activity, sections were incubated in 3% hydrogen peroxide at room temperature for 10 min, followed by washing with PBS (3 times, 5 min each). For renal tissue, an appropriate dilution of primary antibodies against URAT1 or OAT1 was applied, while for hepatic tissue, an appropriate dilution of XOD antibody was used. Sections were incubated with the primary antibodies overnight at 4 °C. After washing with PBS (3 times, 5 min each), sections were incubated with biotinylated rabbit secondary antibodies for 60 min followed by additional PBS washes (3 times, 5 min each). Color development was achieved using 3,3′-diaminobenzidine, and sections were counterstained with hematoxylin to visualize cell nuclei. Images were quantitatively analyzed using ImageJ software.

### 2.12 Western blotting

The expression levels of URAT1, OAT1, Collagen I, α-SMA and XOD proteins in renal and hepatic tissues were determined using Western blotting, which was performed to detect the protein levels in tissue samples (Zhu et al., 2022). Renal and hepatic tissue homogenates were prepared, and proteins were extracted according to the lysis buffer manufacturer’s instructions, with the addition of protease inhibitors. The homogenates were centrifuged at 4 °C and 12,000 rpm for 15 min, and the supernatant was collected. Protein concentrations were quantified using the Bicinchoninic Acid (BCA) Protein Assay Kit following the manufacturer’s protocol. Protein samples were prepared based on the molecular weights of the target proteins, loaded onto gels, and subjected to electrophoresis for separation, followed by transfer to a membrane. The membrane was blocked at room temperature for 60–90 min. After washing with TBST, the membrane was incubated with primary antibodies against URAT1, OAT1, Collagen I, α-SMA or XOD overnight at 4 °C (URAT1, 1:3000; OAT1, 1:3000; Collagen I, 1:1000; α-SMA, 1:3000). The following day, the membrane was washed with TBST and incubated with the corresponding secondary antibody (1:10,000) for 60 min at room temperature. Protein bands were visualized, and their expression levels were quantified using ImageJ software.

### 2.13 RT-qPCR

According to the literature (Wang et al., 2024b), total RNA was extracted from kidney tissue using Trizol Reagent. The primer sequence information is shown in Table 3.

**TABLE 3:** Primer sequence information.

Primer name	Sequences (5′to 3′)	Tm (°C)	Product size (Bp)
GAPDH: F GAPDH: R	CCTCGTCCCGTAGACAAAATG TGAGGTCAATGAAGGGGTCGT	60	133
URAT1: F URAT1: R	CGCTTCCGACAACCTCAAT GAGTTACATACCAGGTCCCACG	60	155
OAT1: F OAT1: R	TGTGCTTCCTAGTCATCAATTCCA CAGGGATGTGCGAATGATTGTA	60	128

### 2.14 Statistical analysis

Data were analyzed using SPSS 23.0 software. Experimental results are expressed as mean ± standard deviation (SD). One-way analysis of variance (ANOVA) was used for comparisons among multiple groups, followed by the Least Significant Difference (LSD) test for pairwise comparisons between groups. Statistical graphs were generated using GraphPad Prism 9 software. The difference was considered statistically significant when P < 0.05.

## 3 Results and analysis

### 3.1 Effect of fermentation on the content of active components in SP

**FIGURE 1:**
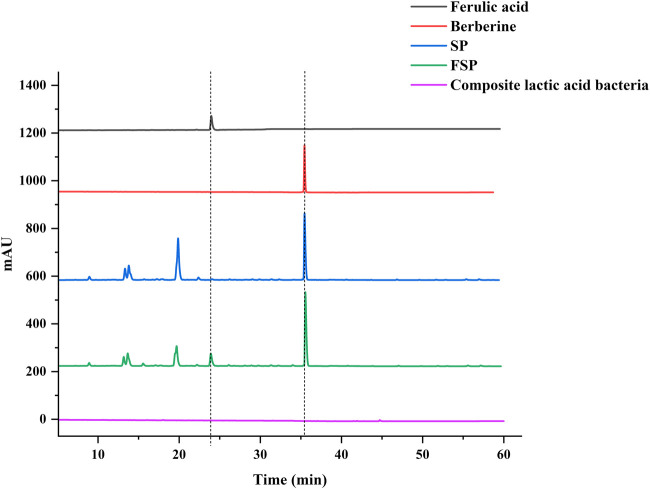
HPLC chromatogram comparison of SP and FSP.

As shown in Table 4, the content of ferulic acid in SP was 0.110 mg/mL, increasing to 0.154 mg/mL in FSP, representing a 40% increase. The content of berberine was 5.225 mg/mL, rising to 6.308 mg/mL, a 20.72% increase. Both active components exhibited increased concentrations following microbial fermentation. The HPLC chromatograms illustrating the changes in active component content of SP and FSP are presented in Figure 1.

**TABLE 4:** Changes of active ingredients in SP and FSP.

Title	Ferulic acid		Berberine	
	Concentration (mg/mL)	Peak area	Concentration (mg/mL)	Peak area
SP	0.110	62.5	5.225	3,293.5
FSP	0.154	913.4	6.308	3,965.8
Improvement rate	40.0%		20.7%	

### 3.2 Single-factor experimental results

**FIGURE 2:**
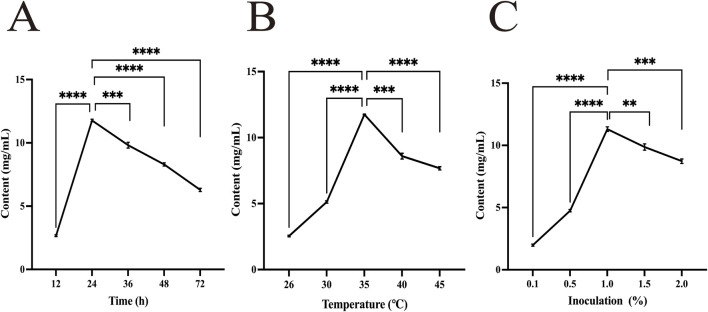
Effect of each variable factor on chemical content. **(A)** Time; **(B)** temperature; **(C)** inoculation. **P < 0.01, ***P < 0.001, ****P < 0.0001.

As shown in Figure 2, under constant conditions with a fermentation time of 24 h, a fermentation temperature of 35 °C, and an inoculation of 1%, the total content of ferulic acid and berberine in FSP reached relatively high levels, recorded at 11.76 mg/mL, 11.74 mg/mL, and 11.30 mg/mL, respectively.

### 3.3 Response surface analysis

#### 3.3.1 Establishment and variance analysis of the quadratic multivariate model equation

The Box-Behnken experimental design and results are presented in Table 5. Statistical analysis of Table 5 using Design Expert 13 software yielded the following regression equation:

$$R=-110.81+1.84A+5.33B+7.21C+0.008\text{AB}+0.03\text{AC}-0.008\text{BC}-0.43A^{2}-0.07B^{2}-3.51C^{2}.$$

**TABLE 5:** Experimental design results of response surface.

Run	A	B	C	R
	Time (h)	Temperature (°C)	Inoculation (%)	(mg/mL)
1	−1	−1	0	2.54
2	0	−1	−1	7.91
3	1	0	−1	5.75
4	0	−1	1	8.79
5	1	1	0	5.62
6	0	0	0	12.12
7	0	1	1	9.92
8	1	0	1	6.61
9	−1	1	0	1.81
10	0	0	0	11.24
11	1	−1	0	4.29
12	−1	0	−1	3.04
13	0	1	−1	9.12
14	0	0	0	11.78
15	0	0	0	11.56
16	−1	0	1	3.15
17	0	0	0	12.19

Variance analysis of the model is shown in Table 6. The P-value <0.0001 indicates that the quadratic multivariate regression model is highly significant, with an F-value of 150.30. The lack-of-fit term was not significant, with an F-value of 1.04. The R^2^ value of 0.9949 demonstrates that 99.49% of the optimized results successfully fit the model, confirming the high reliability of the model.

**TABLE 6:** Response surface analysis results.

Source	Sum of squares	df	Mean square	F value	p-value	Significance
Model	214.82	9	23.87	150.30	<0.0001	Significant
A	17.20	1	17.20	108.30	<0.0001	​
B	1.08	1	1.08	6.80	0.0350	​
C	0.8778	1	0.8778	5.53	0.0510	​
AB	1.06	1	1.06	6.68	0.0362	​
AC	0.1406	1	0.1406	0.8855	0.3780	​
BC	0.0016	1	0.0016	0.0101	0.9229	​
A^2^	164.75	1	164.75	1,037.42	<0.0001	​
B^2^	16.14	1	16.14	101.62	<0.0001	​
C^2^	3.30	1	3.30	20.78	0.0026	​
Residual	1.11	7	0.1588	​	​	Not significant
Lack of fit	0.4880	3	0.1627	1.04	0.4644	​
Pure error	0.6237	4	0.1559	​	​	​
Cor total	215.93	16	​	​	​	​
*R* ^2^	0.9949					
Adj *R* ^2^	0.9593					
CV%	5.32					
AP	32.1513					

**FIGURE 3:**
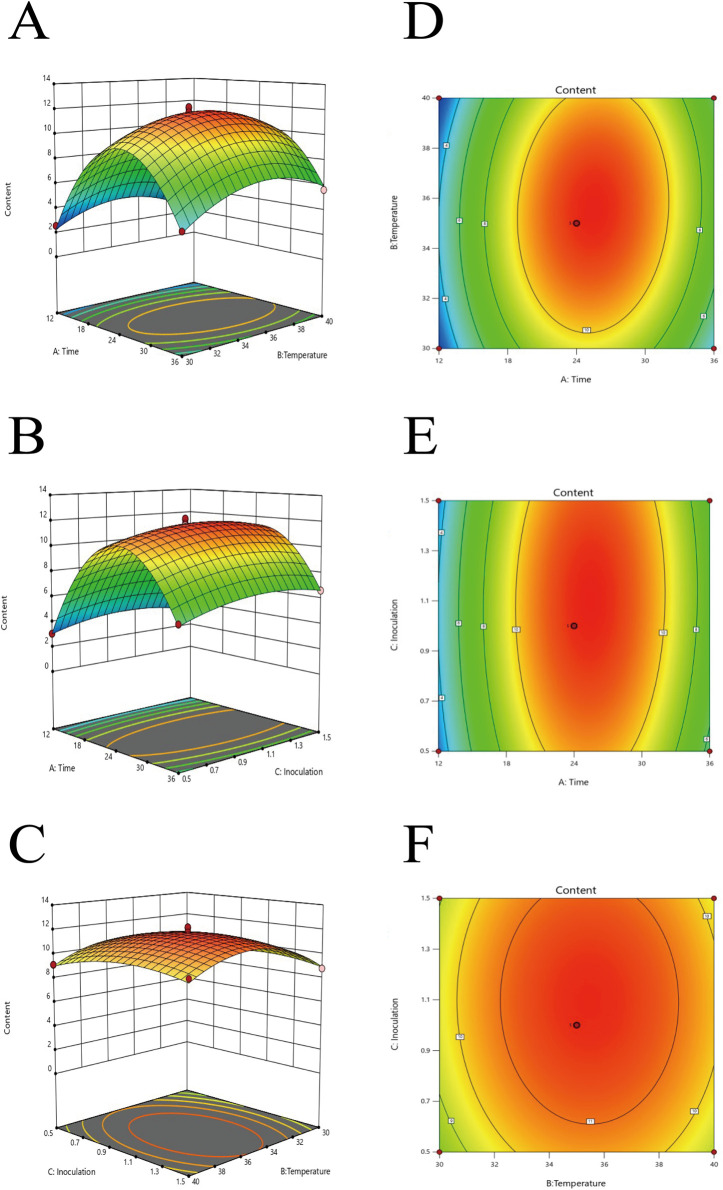
**(A)** Interaction Curve of Time and Temperature; **(B)** Interaction Curve of Time and Inoculation; **(C)** Interaction Curve of Temperature and Inoculation; **(D)** Interaction Contour of Time and Temperature; **(E)** Interaction Contour of Time and Inoculation; **(F)** Interaction Contour of Temperature and Inoculation.

Three-dimensional response surface plots illustrating the effects of two-factor interactions on the total content of active components (ferulic acid and berberine) in FSP broth are shown in Figure 3. As depicted in Figures 3A–C, the contents of ferulic acid and berberine increased with fermentation time and temperature, enhancing the interaction between these two factors. Additionally, the contents increased with higher temperature and inoculation. As shown in Figures 3D–F, the highest total content of ferulic acid and berberine in FSP was achieved at a fermentation time of 24 h, a temperature of 35 °C, and an inoculation of 1%.

#### 3.3.2 Validation experiments

Validation experiments were conducted under the optimized fermentation conditions, with three replicates. The results showed an average total content of ferulic acid and berberine of 11.69 mg/mL. This value closely aligns with the model’s predicted value, confirming the reliability of the fermentation conditions.

### 3.4 Body weight changes of mice in each group

**FIGURE 4:**
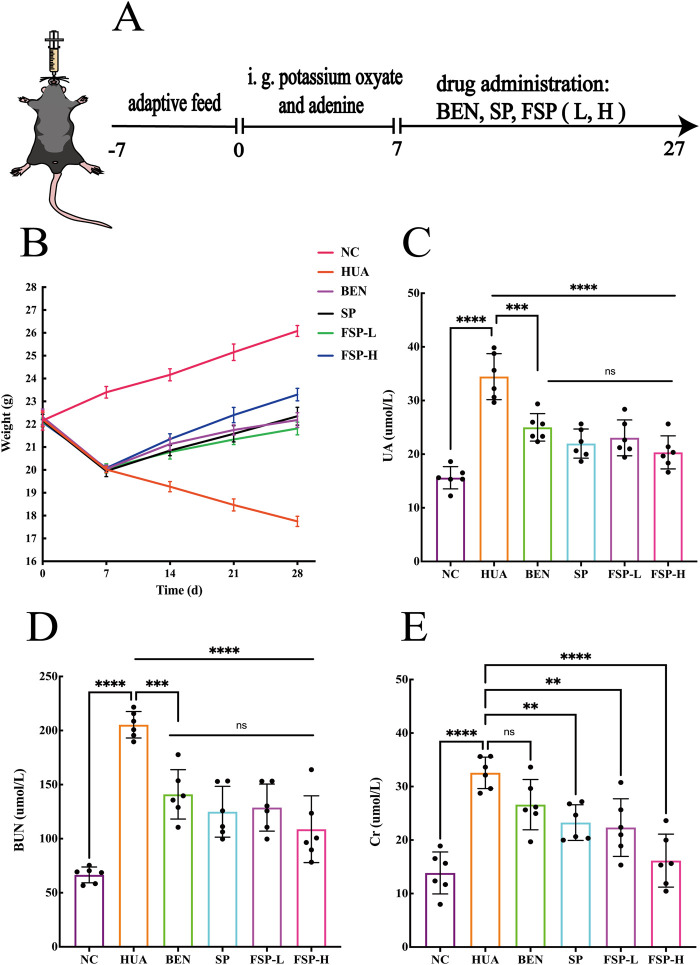
**(A)** Schematic diagram of the animal experiment; **(B)** body weight curve of mice; **(C)** Uric acid (UA) levels of mice; **(D)** Blood urea nitrogen (BUN) levels of mice; **(E)** Creatinine (Cr) levels of mice. ns, not significant; P > 0.05, **P < 0.01, ***P < 0.001, ****P < 0.0001.

Changes in body weight reflect the effects of the drug-induced HUA model. As shown in Figure 4B, during the modeling period, the body weight of mice in all groups exhibited a decreasing trend, with the exception of the NC group. After drug administration, the body weight of mice in all groups increased to varying degrees, except for the model group.

### 3.5 Effects of FSP on UA, BUN and Cr levels

UA is correlated in uric acid metabolism. BUN and Cr are key biomarkers of kidney function. The results (Figures 4C–E) showed that UA, BUN and Cr levels in the HUA group were significantly elevated compared to the NC group (P < 0.0001). Compared to the HUA group, all treatment groups exhibited varying degrees of reduction in UA, BUN and Cr levels. Notably, the FSP-H group demonstrated the most significant decrease in UA, BUN and Cr levels (P < 0.0001), indicating a potent uric acid-lowering effect and improved glomerular filtration function in hyperuricemic mice.

### 3.6 Effect of FSP on kidney function

**FIGURE 5:**
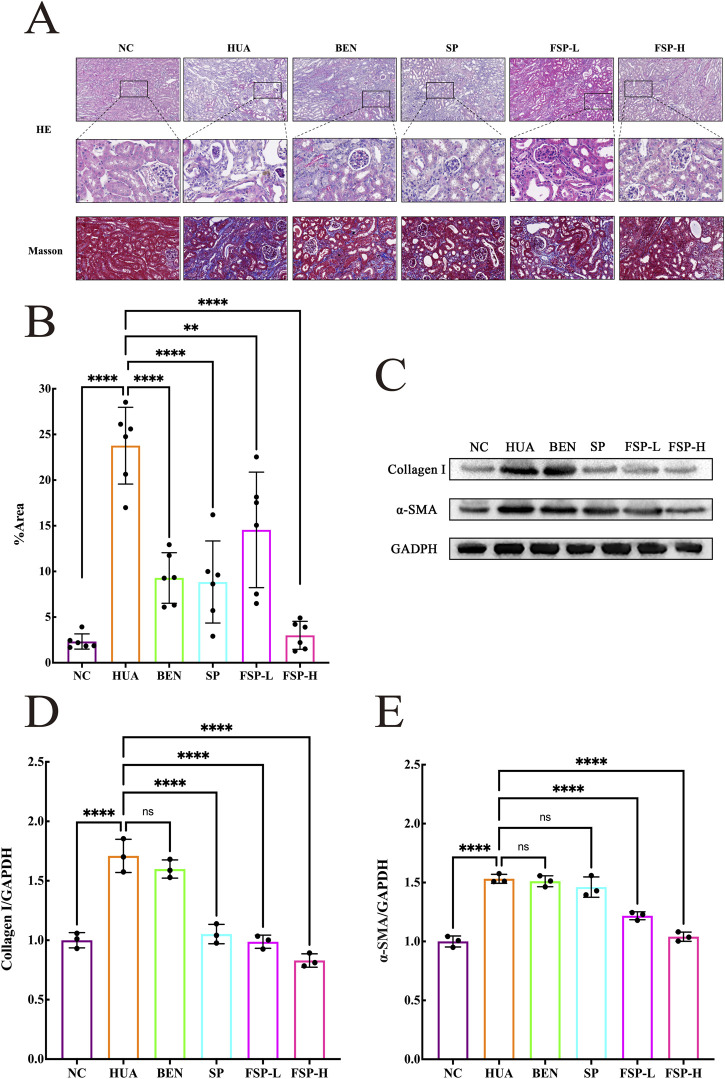
**(A)** HE staining and Masson’s trichrome staining of renal tissue; **(B)** Analysis of the area occupied by positive renal fibrosis; **(C)** The representation of the Western blotting results of collagen I and α-SMA in renal tissue; **(D)** Statistic quantification of collagen I protein expression level in renal tissue; **(E)** Statistic quantification of α-SMA protein expression level in renal tissue. ns, not significant; P > 0.05, **P < 0.01, ****P < 0.0001.

The results of the HE, Masson staining and Western blotting of renal tissue were displayed in Figure 5. HE staining revealed that the renal tissue of the NC group exhibited a uniform, dense connective tissue capsule on the surface. Glomeruli showed consistent cellularity and matrix, while renal tubular epithelial cells appeared plump and rounded with well-arranged brush borders. No significant interstitial hyperplasia or inflammatory cell infiltration was observed. In the HUA group, the arrangement of renal tubular cells was disordered, accompanied by glomerular sclerosis and tubular dilation. Notable vacuolation was observed in both glomeruli and tubules, with cells displaying extensive vacuolar degeneration. In addition, a large amount of collagen fiber deposition was observed in the renal tubules of the HUA group, and the protein expression levels of collagen I and α-SMA were significantly higher than those in the NC group (P < 0.0001). These findings indicated that the renal structure of the HUA group had undergone significant pathological alterations. All treatment groups showed varying degrees of improvement in renal histopathological features. Notably, the FSP-H group exhibited the most significant amelioration of renal injury, and the protein expression levels of collagen I and α-SMA were markedly lower than those in the HUA group (P < 0.0001), returning to normal levels. These findings suggested that FSP more effectively mitigates kidney damage associated with hyperuricemia.

### 3.7 Effects of FSP on liver function

**FIGURE 6:**
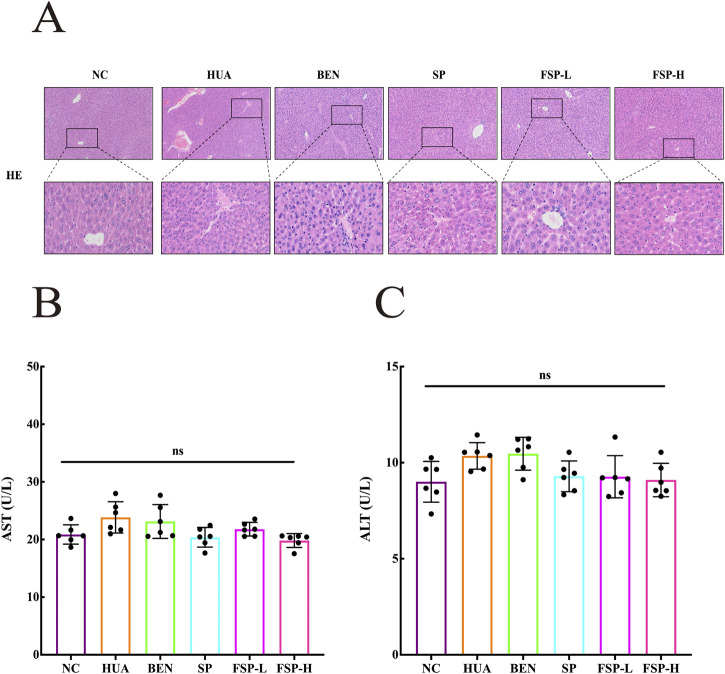
**(A)** HE staining of hepatic tissue; **(B)** Effect of FSP on serum AST; **(C)** Effect of FSP on serum ALT. ns: not significant, P > 0.05.

As indicated in Figure 6A, in the HUA group and BEN group, hepatocytes displayed proliferation, with unclear lobular architecture, disordered hepatic cord arrangement, and significant erythrocyte accumulation in the hepatic sinusoids. In contrast, the SP, FSP-L and FSP-H groups exhibited clear lobular architecture, orderly hepatic cord arrangement, and no evident erythrocyte accumulation.

ALT and AST are commonly used as important indicators of liver function. As shown in Figures 6B,C, compared with the NC group, the serum ALT and AST levels in the HUA group and the BEN group were increased, with values of 23.84 ± 0.27 U/L and 23.13 ± 0.29 U/L, 10.35 ± 0.29 U/L and 10.47 ± 0.25 U/L, respectively. While those in the other treatment groups returned to normal levels, with no significant difference (P > 0.05, ns). These findings indicate that neither SP nor FSP induces hepatotoxicity in mice.

### 3.8 Effect of FSP on renal protein expression of URAT1 and OAT1

**FIGURE 7:**
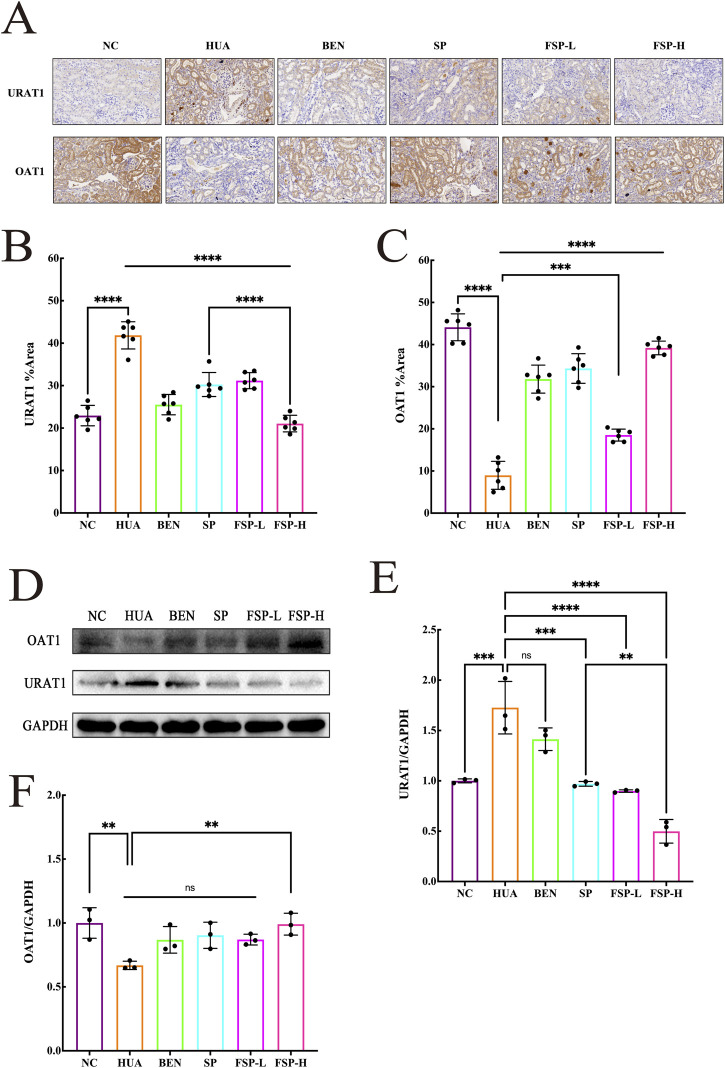
**(A)** Immunohistochemical results of URAT1 and OAT1 in renal tissue (400×); **(B)** The quantification of URAT1 immunohistochemistry staining; **(C)** The quantification of OAT1 immunohistochemistry staining; **(D)** The representation of the Western blotting results of URAT1, OAT1 in renal tissue; **(E)** Statistic quantification of URAT1 protein expression level in renal tissue; **(F)** Statistic quantification of OAT1 protein expression level in renal tissue. ns: not significant, P > 0.05, **P < 0.01, ***P < 0.001, ****P < 0.0001.

The protein expressions of key transporters URAT1 and OAT1 in the kidney were determined by Immunohistochemistry and Western blot analysis (Figure 7). Compared with the NC group, the HUA group exhibited significantly increased expression of URAT1, with a larger positive staining area, while OAT1 expression was markedly reduced, with a smaller positive staining area (P < 0.0001). Compared to the HUA group, all treatment groups showed varying degrees of reduction in the positive staining areas for URAT1, with the FSP-H group demonstrating the most pronounced decrease (P < 0.0001). These results indicated that the FSP-H group effectively inhibited urate reabsorption, promoted uric acid excretion, thereby reducing purine synthesis and lowering systemic uric acid levels.

### 3.9 Effect of FSP on gene level of URAT1 and OAT1

**FIGURE 8:**
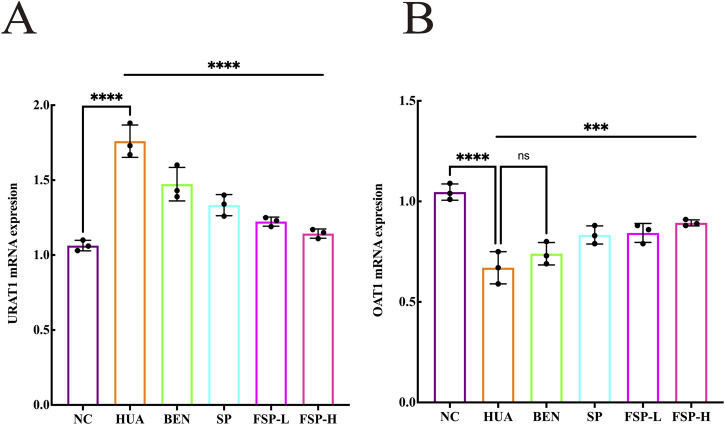
**(A)** Effect of FSP on renal mRNA expression of URAT1; **(B)** Effect of FSP on renal mRNA expression of OAT1. ns, not significant; P > 0.05, ***P < 0.001, ****P < 0.0001.

RT-qPCR was used to evaluate the effect of FSP on the mRNA expression of renal URAT1 and OAT1 (Figure 8). Compared with the NC group, the expression of mURAT1 was significantly increased, while that of mOAT1 was significantly decreased in the HUA group (P < 0.001). Compared with the HUA group, FSP-H significantly downregulated the expression of mURAT1 and upregulated the expression of mOAT1 (P < 0.001, P < 0.001).

### 3.10 Effects of FSP on hepatic XOD activity and protein expression

**FIGURE 9:**
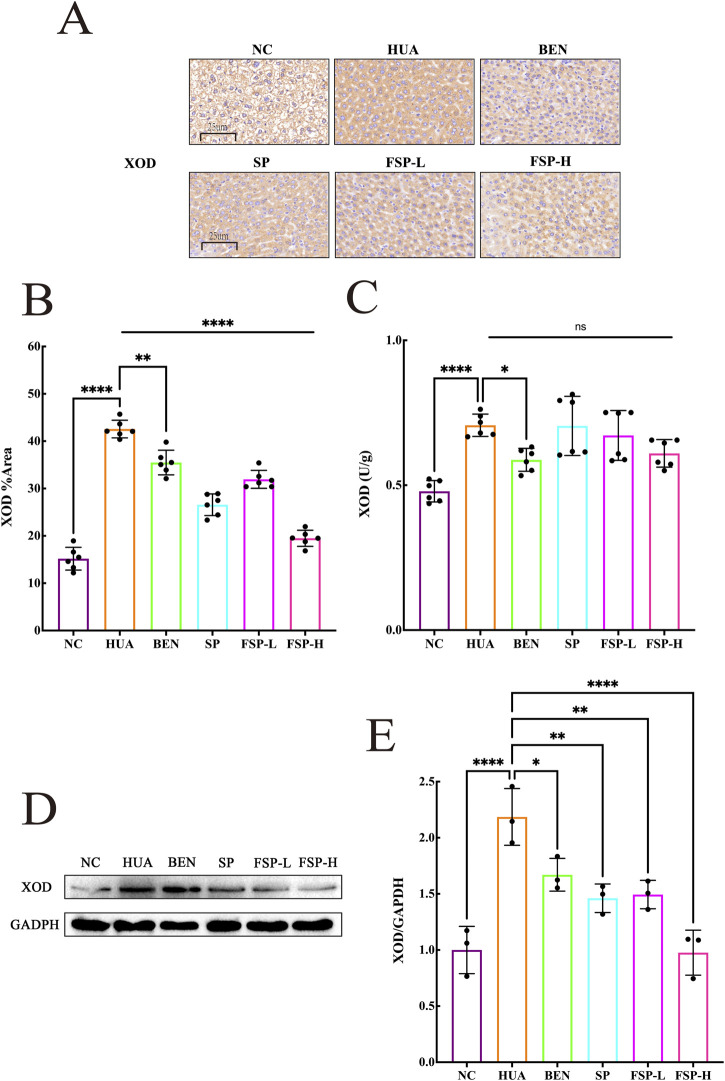
**(A)** Immunohistochemical results of XOD in hepatic tissue (400×); **(B)** The quantification of XOD immunohistochemistry staining; **(C)** Xanthine oxidase (XOD) in liver of mice; **(D)** The representation of the Western blotting results of XOD in hepatic tissue; **(E)** Statistic quantification of XOD protein expression level in hepatic tissue. ns: not significant, P > 0.05, *P < 0.05, **P < 0.01, ****P < 0.0001.

XOD is a key enzyme involved in uric acid production. Effect of FSP on hepatic XOD activity was shown in Figure 9C. The levels of hepatic XOD in the HUA group was significantly elevated compared to the NC group (P < 0.0001). While the SP group showed only a modest reduction in XOD levels relative to the HUA group, both FSP-L and FSP-H groups exhibited more pronounced decreases. These findings indicated that FSP could suppress XOD activity, thereby inhibiting the oxidation of xanthine to uric acid.

The effects of FSP on XOD protein expression in hyperuricemic mice were investigated by immunohistochemistry and Western blot assays. As shown in Figure 9, compared to the NC group, the HUA group exhibited significantly increased protein expression of hepatic XOD, with a larger positive staining area. Compared to both the HUA group and the SP group, the FSP-H group demonstrated a significant reduction in XOD protein expression, and decreased the proportion of positive area (P < 0.0001). These results indicated that the FSP-H group effectively suppressed the catalysis of hypoxanthine by XOD, reduced purine production, and thereby decreased the uric acid level in the organism.

## 4 Discussion

Fermented traditional Chinese medicines with microbes uses enzymes from the microbes to drive chemical reactions under mild conditions. This can increase effectiveness and reduce side effects (Wang et al., 2020). We investigated the effects of composite lactic acid bacteria-fermented SP on its active components, and further explored whether FSP works better than SP against hyperuricemia and elucidated the underlying molecular mechanisms. In this study, SP was fermented with composite lactic acid bacteria, resulting in a significant increase in the content of active components, such as ferulic acid and berberine.

HUA is a secondary renal disorder caused by an imbalance between uric acid production and excretion (Ke et al., 2024). High uric acid levels are a major risk factor for gout, arthritis, hypertension, diabetes, and kidney disease (Kuwabara et al., 2018). Adenine, a building block of uric acid, is converted by XOD into an insoluble substance that can induce renal damage (Khan et al., 2022). Potassium oxonate, structurally similar to uric acid, competitively inhibits urate oxidase, thereby suppressing uric acid excretion (Liu et al., 2023). To closely mimic the human condition of hyperuricemia, which lacks functional urate oxidase (Guo et al., 2015), this study established a mouse model using oral administration of 200 mg/kg potassium oxonate combined with 75 mg/kg adenine. Compared to the NC group, the HUA group exhibited significantly elevated UA, BUN, Cr, and hepatic XOD levels (P < 0.0001). The results also showed mild damage in renal and hepatic tissues, including inflammatory cell infiltration and disordered cellular architecture. This is consistent with the results obtained in reference (Hua et al., 2023), confirming successful model establishment. Compared to the HUA and SP groups, the FSP-H group significantly reduced UA and BUN levels (P < 0.0001, P < 0.001) and showed a trend toward restoring XOD levels to normal. Renal histopathological analysis revealed varying degrees of amelioration in kidney damage across all treatment groups, indicating a protective effect on renal function. Liver histopathology showed similar pathological changes in the BEN and HUA groups, suggesting potential hepatotoxicity associated with benzbromarone. In contrast, both SP and FSP groups exhibited improved liver histopathology, indicating no significant hepatotoxicity.

URAT1 is the first protein identified as being involved in uric acid transport, and it is currently the primary protein known to mediate uric acid reabsorption (Li et al., 2013). The upregulation of URAT1 impairs the body’s excretion of uric acid, leading to elevated serum uric acid levels. Clinically, drugs such as probenecid and benzbromarone reduce uric acid reabsorption by inhibiting URAT1 expression (Taniguchi et al., 2019). OAT1 is located on the basement membrane of proximal renal tubular epithelial cells. OAT1 is the main transporters responsible for the renal excretion of uric acid (Uwai et al., 2004). Increased OAT1 expression promotes uric acid excretion, thereby lowering uric acid levels (Vávra et al., 2022). XOD is one of the key rate-limiting enzymes in the biosynthesis of uric acid in the body and is located in the cytoplasm of liver cells. Purine metabolism in the liver produces uric acid through a two-step reaction: first, XOD oxidizes xanthine to xanthone. Then, xanthone is further oxidized to uric acid. The upregulation of XOD helps inhibit xanthine from catalyzing hypoxanthine and reduces purine production, thereby lowering the body’s uric acid levels (Han et al., 2020). This study found that the FSP-H group significantly reduced URAT1 protein expression while upregulating OAT1 expression, and supressed XOD protein expression compared to the HUA and SP groups. Our findings are in agreement with the work of Han et al. (2020). These results suggested that FSP more effectively inhibited uric acid reabsorption, promoted uric acid excretion, and suppressed uric acid synthesis compared to SP.

Although this study demonstrated that FSP effectively alleviated HUA and renal injury, several limitations should be noted. The present study cannot fully distinguish the individual contributions of lactic acid bacteria and fermented herbal components. Experiments were only performed in mice, without validation in rats or large animal models. Only short-term efficacy was observed, lacking long-term safety and effectiveness evaluation. Furthermore, accumulating evidence has demonstrated that gut microbiota plays a core driving role in the early progression of various metabolic diseases including diabetes and hyperuricemia (Dong et al., 2025). However, the present study did not thoroughly clarify the *in vivo* metabolic processes of key active ingredients after fermentation, nor systematically reveal the regulatory role of gut microbiota in its pharmacological effects. Future studies should verify efficacy and safety in larger animal models to support clinical translation. Using metabolomics, network pharmacology, and microbiomics, the active components, metabolic pathways, and regulatory effects on gut microbiota can be further explored. This will help clarify the overall molecular mechanism and provide a scientific basis for developing safe, gut microbiota-targeted natural medicines.

## 5 Conclusion

Compared to the SP, FSP with a 1% composite lactic acid bacteria inoculum at 35 °C for 24 h resulted in an increased content of ferulic acid and berberine. Besides, FSP is more conducive to lowering uric acid levels in HUA mice, and the mechanism involved up-regulating OAT1 to enhance uric acid excretion, down-regulating URAT1 to inhibit uric acid reabsorption, and suppressing XOD to reduce uric acid production. Moreover, FSP had a very good safety profile at all tested doses.

## Data Availability

The original contributions presented in the study are included in the article/supplementary material, further inquiries can be directed to the corresponding authors.
